# Integration of photonic nanojets and semiconductor nanoparticles for enhanced all-optical switching

**DOI:** 10.1038/ncomms9097

**Published:** 2015-08-28

**Authors:** Brandon Born, Jeffrey D. A. Krupa, Simon Geoffroy-Gagnon, Jonathan F. Holzman

**Affiliations:** 1 School of Engineering, The University of British Columbia, Okanagan campus, Kelowna, British Coloumbia, Canada, V1V 1V7

## Abstract

All-optical switching is the foundation of emerging all-optical (terabit-per-second) networks and processors. All-optical switching has attracted considerable attention, but it must ultimately support operation with femtojoule switching energies and femtosecond switching times to be effective. Here we introduce an all-optical switch architecture in the form of a dielectric sphere that focuses a high-intensity photonic nanojet into a peripheral coating of semiconductor nanoparticles. Milli-scale spheres coated with Si and SiC nanoparticles yield switching energies of 200 and 100 fJ with switching times of 10 ps and 350 fs, respectively. Micro-scale spheres coated with Si and SiC nanoparticles yield switching energies of 1 pJ and 20 fJ with switching times of 2 ps and 270 fs, respectively. We show that femtojoule switching energies are enabled by localized photoinjection from the photonic nanojets and that femtosecond switching times are enabled by localized recombination within the semiconductor nanoparticles.

## Abstract (web summary)

The terabit-per-second rates of optical fibres exceed the capability of electronics, but all-optical switches are needed to alleviate the bottleneck in these networks. Here, Born *et al.* describe such a switch using dielectric spheres, yielding femtojoule switching energy and femtosecond switching times.

## Graphical abstract

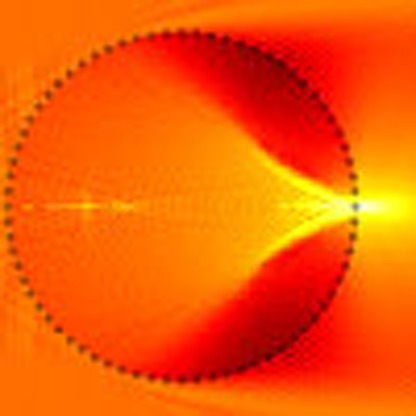

Logic operations are at the core of data processing, and switching is the key mechanism for logic operations. Switching is typically realized via electronics, with transistors relaying the flow of data, but there is pressure to introduce switches that can operate at optical fibre transmission rates^1^,^2^. The terabit-per-second rates of optical fibres exceed the gigabit-per-second rates of electronics—and the all-optical switch (AOS) has been proposed to alleviate the ensuing optoelectronic bottleneck in front-end networks^3^,^4^.

The AOS is a photonic analogue to the electronic transistor, as it enables nonlinear mixing of optical beams, but all-optical switching must be implemented with consideration to demands for low switching energies and ultrafast switching times^5^,^6^,^7^. Many AOS technologies have been proposed in contemporary literature to address these demands^7^,^8^,^9^,^10^,^11^.

Early AOS studies focused on the development of semiconductor materials for ultrafast switching times—ideally on a femtosecond level. Charge-carrier photoinjection and recombination were applied in semiconductors having simple planar topologies, that is, bulk wafers, so the all-optical switching times were limited by bulk recombination lifetimes. It was later found that semiconductors with ultrashort lifetimes could establish ultrafast switching between coincident control (pump) and signal (probe) beams. For example, Ganikhanov *et al.*^12^ and Gupta *et al.*^13^ demonstrated ultrafast all-optical switching using ion-implanted and low-temperature-grown GaAs, respectively. Overall, ultrafast switching times could be achieved with these material systems, but the use of simple focusing in semiconductors was found to limit the beam intensities, necessitating high switching energies^14^.

More recent AOS studies have focused the development of device geometries for low switching energies—ideally on a femtojoule level. Increased beam intensities, and enhanced mixing of beams, have been introduced through many (typically resonant) devices. Almeida *et al.*^9^ demonstrated micrometer-scale AOS devices as ring resonators. Nozaki *et al.*^10^ demonstrated nanometer-scale AOS devices as photonic crystal resonators. Volz *et al.*^11^ demonstrated atomic-scale AOS devices as quantum dots. Such resonant devices support low switching energies, in general, but their implementation must consider the prolonged switching times that are inherent to resonant cavity lifetimes^15^.

In this study, the demands of all-optical switching, being AOS activation with femtojoule switching energies and AOS recovery with femtosecond switching times, are addressed through the development of a non-resonant AOS architecture. A nanojet focal geometry is introduced, in the form of a dielectric sphere, to focus high-intensity photonic nanojets into a semiconductor nanoparticle material system that coats the sphere. It is found that the localized photoinjection of charge carriers by the nanojet supports AOS activation with femtojoule switching energies, and the localized recombination of charge carriers in the semiconductor nanoparticles supports AOS recovery with femtosecond switching times. A milli-scale AOS architecture is demonstrated—in support of emerging optical fibre front-end systems, such as optical time-division multiplexing^16^, orthogonal frequency-division multiplexing^17^, and other all-optical multiplexing systems. A micro-scale AOS architecture is demonstrated—in support of future on-chip optical processors, such as network-on-a-chip systems^3^.

## Results

### Semiconductor charge-carrier dynamics

Semiconductor charge carriers that are photoinjected by a pump beam can be used to modulate a coincident probe beam. The pump-induced modulation to the probe beam's total transmission, *T*, is a result of perturbations to the surface transmission, *T*_s_, and bulk transmission, *T*_b_. The result is seen as a transient on the total differential transmission, Δ*T*(*t*)/*T*≈Δ*T*_s_(*t*)/*T*_s_+Δ*T*_b_(*t*)/*T*_b_, with opposing polarities for the surface and bulk contributions. According to Drude theory, the pump-induced generation of the charge-carrier density, *N*(*z*,*t*), increases the differential surface transmission, Δ*T*_s_(*t*)/*T*_s_, and decreases the differential bulk transmission, Δ*T*_b_(*t*)/*T*_b_. This occurs because *N*(*z*,*t*) decreases the refractive index near the surface, Δ*n*_s_(*t*), and increases the absorption coefficient in the bulk, Δ*α*_b_(*t*), which modulates the total differential transmission according to^18^


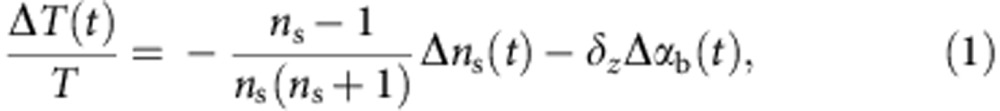


where *n*_s_ is the refractive index near the surface, and *δ*_*z*_ is the photoinjection depth of charge carriers into the bulk.

Both Δ*n*_s_(*t*) and Δ*α*_b_(*t*) evolve in proportion to the charge-carrier density, *N*(*z*,*t*), which varies through time, *t*, along the normal dimension to the semiconductor surface, *z*, according to


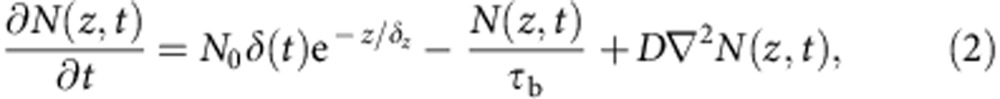


where the three terms on the right-hand side characterize the respective processes of photoinjection, recombination, and diffusion. Photoinjection from an ultrashort laser pulse is applied by way of the time-varying delta function, *δ*(*t*). The initial distribution for *N*(*z*,*t*) is an exponential decay into the semiconductor, with an initial charge-carrier density of *N*_0_ at the surface and a 1/e photoinjection depth of *δ*_*z*_ into the bulk. The photoinjection depth, *δ*_*z*_, is defined here as a general parameter that can be less than or equal to the semiconductor's penetration depth. Given a suitable focal geometry, with tight focusing and a short depth of focus penetrating into the semiconductor, *δ*_*z*_ can be made to be less than the semiconductor's penetration depth. The boundary condition for *N*(*z*,*t*) at the semiconductor surface adheres to *D*∂*N*(*z*=0,*t*)/∂*z*=*S*_v_*N*(*z*=0,*t*), for a diffusion coefficient, *D*, and surface recombination velocity, *S*_v_. Given a suitable material system, with a high density of surface states, this boundary condition can reduce the overall charge-carrier lifetime, *τ*, to a value below the bulk charge-carrier lifetime, *τ*_b_.

Localized photoinjection can enable AOS activation with femtojoule switching energies, by minimizing the pump pulse energy, *E*_p_, that is needed to establish the initial charge-carrier density, *N*_0_=*E*_p_*η*/(*ħω*_p_*V*), in the first term of equation (2). Here η is the internal quantum efficiency, *ħ* is the reduced Planck's constant, *ω*_p_ is the angular frequency of the pump beam, and *V*≈*A*_*φ*_*δ*_*z*_ is the photoinjection volume set by the pump beam's cross-sectional photoinjection area, *A*_*φ*_, and photoinjection depth, *δ*_*z*_. In this study, localized photoinjection is applied by way of a nanojet focal geometry, to yield a reduced focal spot size, that is, photoinjection area, and reduced depth of focus, that is, photoinjection depth. This leads to reduced photoinjection volumes and high initial charge-carrier densities.

Localized recombination can enable AOS recovery with femtosecond switching times, by promoting surface recombination and thus minimizing the overall charge-carrier lifetime, *τ*, as a result of equation (2) and its boundary condition. The charge-carrier lifetime simplifies to the recombination rate relation, 1/*τ*=1/*τ*_b_+*S*_v_*R*. An increase in the surface recombination velocity, *S*_v_, or the specific surface area, that is, surface-to-volume ratio, *R*, has *τ* decrease below the bulk charge-carrier lifetime, *τ*_b_. Our prior work has shown that nanoscale cylindrical^19^ and spherical^20^ semiconductor forms exhibit this trend, as an increased surface-to-volume ratio, *R*, increases the surface state density and decreases the charge-carrier lifetime. In this study, localized recombination is applied by way of a nanoparticle material system. It is typically advantageous to apply both a material system with an increased surface state density and a focal geometry that localizes photoinjection in the region of high surface state density—and this two-fold approach is taken here.

The all-optical switching results of this study are defined with respect to nominal tests using a standard focal geometry with a microscope objective and targets comprised of the well-established semiconductors, semi-insulating GaAs, float-zone Si and 6H-SiC, with respective wafer thicknesses of 350, 280 and 330 μm. These semiconductors are particularly advantageous for this study, given their wide range of bulk lifetimes and surface recombination velocities.

**Figure 1:**
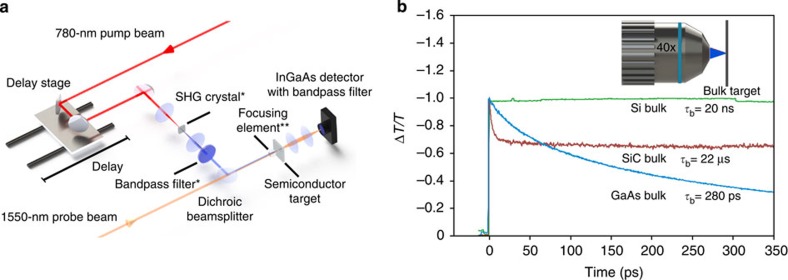
Pump–probe experimental set-up and nominal AOS results. (**a**) Schematic of the pump–probe experimental set-up. A 780-nm pump beam is used for the GaAs and Si targets and a 390-nm pump beam is used for the SiC target. A 1,550-nm probe beam is used for all the targets, and it is re-collimated and focused on the InGaAs detector using two 25-mm-focal length lenses. *A second harmonic generation (SHG) crystal and bandpass filter are placed in the beam path to form the 390-nm pump beam (at a conversion efficiency of 10%). ^**^The focusing element takes the form of a microscope objective or dielectric spheres. (**b**) Experimental differential transmission of the probe beam, Δ*T/T*, is shown normalized versus time, for pump photoinjection of the bulk Si, SiC and GaAs targets, using a microscope objective as the focusing element, as depicted in the inset.

Figure 1a shows the pump–probe experimental set-up, with the corresponding details given in the Methods section. The GaAs and Si targets use pump and probe pulses with respective wavelengths of 780 and 1,550 nm. The SiC target uses pump and probe pulses with respective wavelengths of 390 and 1,550 nm.

Figure 1b shows the experimental differential transmission, Δ*T*/*T*, for the three semiconductor targets. The negative signal polarities indicate that pump-induced modulation to the probe beam is dominated by absorption in the bulk for all three semiconductor targets, which agrees with the observed long bulk lifetimes. GaAs exhibits a picosecond-scale bulk lifetime of *τ*_b_=280 ps, Si exhibits a nanosecond-scale bulk lifetime^21^ of *τ*_b_=20 ns and SiC exhibits a microsecond-scale bulk lifetime^22^ of *τ*_b_=20 μs. The fast transient at the onset of the SiC response is due to charge-carrier scattering. Details on the SiC charge-carrier dynamics are given in the Methods section. All three materials exhibit unacceptably slow recovery times for AOS operation, as minimal contributions are seen from surface recombination in the GaAs, Si and SiC targets, despite their wide range of surface recombination velocities, *S*_v_=1.2 × 10^4^ m s^–1^, *S*_v_=500 m s^–1^ and *S*_v_=4,000 m s^–1^, respectively^20^,^23^.

### The nanojet focal geometry

Localized photoinjection is considered in this section, for enhanced AOS activation, by way of a nanojet focal geometry. A high-intensity photonic nanojet^24^ can be formed by focusing through an appropriately designed dielectric sphere^25^,^26^. The tight transverse constriction and shallow protrusion formed by photonic nanojet focusing, just beyond the sphere, are well suited to the application of localized photoinjection into a peripheral semiconductor. The resulting small photoinjection area, *A*_*φ*_, and depth, *δ*_*z*_, of the pump beam in the semiconductor establish a reduced photoinjection volume, *V*≈*A*_*φ*_*δ*_*z*_. This leads to a large initial charge-carrier density, *N*_0_, and a small charge-carrier lifetime, *τ*, if the photoinjection is preferentially applied at a semiconductor surface. The nanojet focal geometry must be implemented with careful consideration to the properties of the sphere, however, as there is an inherent relationship between the photonic nanojet's intensity and the sphere's diameter, *d*, and refractive index, *n*. With this in mind, the properties of the sphere that yield the optimal nanojet are studied by way of theoretical analyses across two regimes of scale.

**Figure 2:**
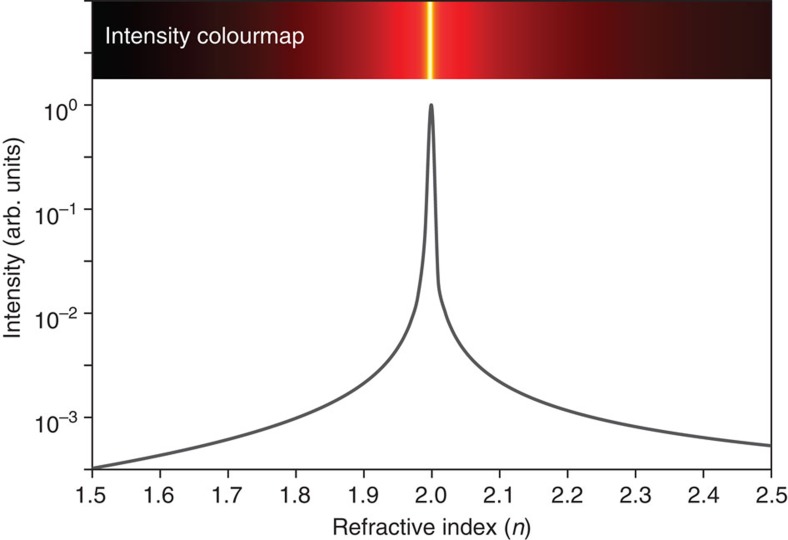
Photonic nanojet intensity in the milli-scale regime. The theoretical intensity of a photonic nanojet, calculated with Ray theory at the exit interface of a dielectric sphere, is shown as a function of the sphere's refractive index, *n*. An intensity colourmap of Ray theory simulations in the milli-scale regime (for diameters of *d*>100 μm) is shown at the top of the figure.

In the milli-scale regime, with sphere diameters that are much larger than the wavelength, focusing is well-described by Ray theory. Figure 2 shows the results from Ray theory applied to the spherical geometry. The intensity at the exit interface of the sphere, intersecting with the optical axis, is displayed as a function of the sphere refractive index, 1.5<*n*<2 for diameters *d*>100 μm. The curve shows that high-intensity focusing at the exit interface of the sphere is brought about the refractive index of *n*≈2. An intensity colourmap for this milli-scale regime is generated from the Ray theory curve and is shown at the top of the figure. The peak intensity for *n*≈2 is shown in white. These findings agree with limiting case of a ball lens in the thick-lens formula^27^, which predicts focusing of light rays at the exit interface for *n*≈2.

**Figure 3:**
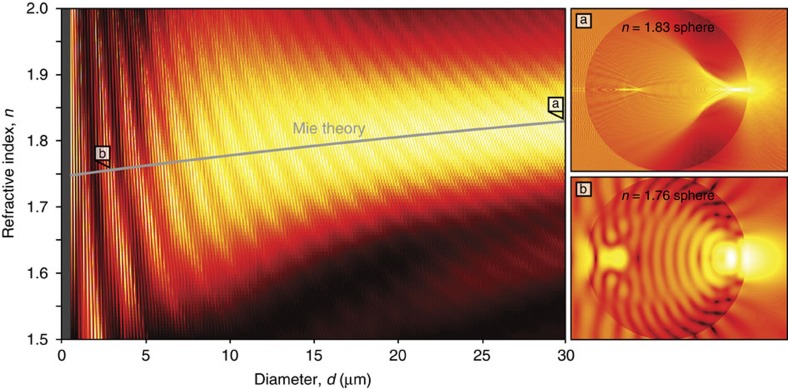
Photonic nanojet intensity in the micro-scale regime. The theoretical intensity of a photonic nanojet, calculated with Mie theory at the exit interface of a dielectric sphere, is shown as a function of the sphere's refractive index, *n*, and diameter, *d*, for pump photoinjection at a wavelength of 780 nm. A trendline for the maximum intensity is shown as a solid grey curve. An intensity colourmap of Mie theory simulations in the micro-scale regime is shown on the left side of the figure—being the result of over a hundred thousand individual Mie theory simulations. The maximum (white) intensity is normalized for all *n* with constant *d*. Select 3D Mie theory simulations, with logarithmic intensities, are shown in the figure insets (**a**) a sphere with *d*=30 μm and *n*=1.83, and (**b**) a sphere with *d*=3 μm and *n*=1.76.

In the micro-scale regime, with sphere diameters that are comparable to the wavelength, it is necessary to implement a rigorous three-dimensional electromagnetic analysis. This can be done efficiently for a spherical geometry using Mie theory. Figure 3 shows the results from Mie Theory analyses applied to the spherical geometry. The work presented is based on the algorithm^26^ of Lecler *et al.* Mie theory simulations are carried out to identify the maximum intensities at the exit interface of the sphere, intersecting with the optical axis, as a function of the sphere refractive index, *n*, and diameter, *d*. The full distribution of intensities from Mie theory is shown for the micro-scale regime as an intensity colourmap map in Fig. 3 for *d*<30 μm and 1.5<*n*<2. The trendline for maximum intensities is shown as a solid blue curve. The curve rises from a low refractive index at small diameters, *n*≈1.75, toward the Ray theory result at large diameters, *n*≈2. The highest intensities are seen as a broad band of intensities that peak at *n*≈1.75 for small *d* then rise toward *n*≈2 for sufficiently large *d*, where the Mie theory and Ray theory results merge.

Given the optical responses of Fig. 3 and the requirements for all-optical switching, it is worth commenting on the potential for contributions from resonance and spherical aberration.

Resonance manifests itself as two patterns of interferometric fringes in the intensity colourmap map for the micro-scale regime in Fig. 3. The first fringe pattern comes about from longitudinal modes that resonate along the optical axis and form the steep high-spatial-frequency fringes in the intensity map. The second fringe pattern comes about from coupled longitudinal and whispering-gallery modes^28^, that exhibit interferometric beating along the perimeter and form the sloped low-spatial-frequency fringes in the intensity map. It is worth noting that resonance can be avoided, however, if there is a desire for reduced sensitivity to structural and thermal fluctuations, by applying laser pulses with sufficiently short durations (being much shorter than the cavity lifetime) or by applying spheres with sufficiently large diameters (being at the upper end of the micro-scale regime or anywhere within the milli-scale regime).

Spherical aberration manifests itself as imperfect focusing at the exit interface of the sphere. This leads to an increased demand on the switching energy, as the incident beam power must be increased to compensate for the reduced intensity. Note that spherical aberration can be compensated, to some degree, in that a sphere, being approximated as a plane-parallel plate between two plano-convex lenses, will have spherical aberration be over-corrected by the plane-parallel plate and under-corrected by the lenses^29^. In general, spherical aberration decreases as the refractive index increases. Using Ray theory, it can be shown that a sufficiently high refractive index sphere, with *n*≈2, has a 42% reduction in the (transverse) spherical aberration, compared with that of a sphere with a refractive index of *n*≈1.5.

**Figure 4:**
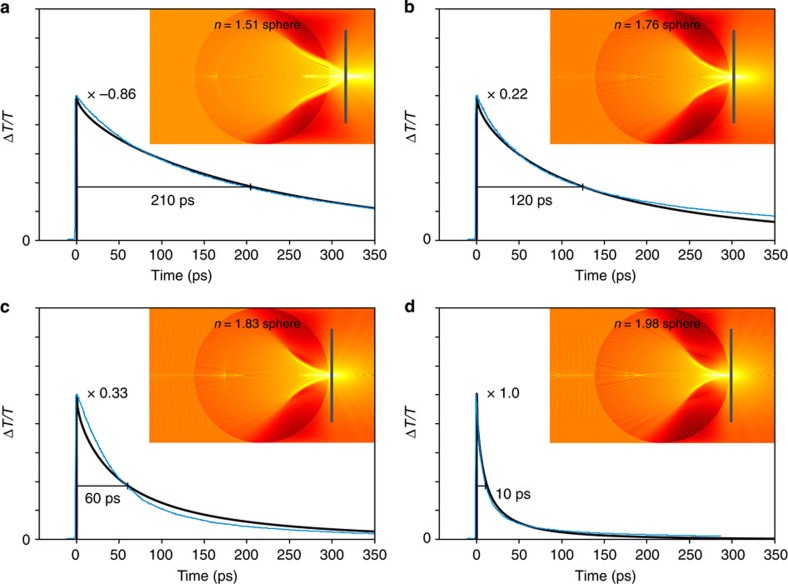
All-optical switching with varying sphere refractive indices. Theoretical and experimental differential transmission curves of the probe beam, Δ*T/T*, are shown normalized versus time, for photoinjection into a GaAs target, using spheres with a diameter of *d*=2 mm and refractive indices of (**a**) *n*=1.51, (**b**) *n*=1.76, (**c**) *n*=1.83 and (**d**) *n*=1.98±0.02. The differential transmission results are shown normalized, with respect to the results in (**d**) and the relative scaling factors are labelled in the figures. The figures include theoretical curves from the Drude/charge-carrier dynamical model based on equations (1) and (2). Mie theory simulations are shown in the insets to illustrate the varying focal conditions of the coincident pump and probe beams.

The measured differential transmission, Δ*T*/*T*, is shown in Fig. 4, as a function of time, for the spheres having refractive indices of (a) *n*=1.51, (b) *n*=1.76, (c) *n*=1.83 and (d) *n*=1.98. The experimental curves are shown with corresponding theoretical curves, having been generated by a Drude/charge-carrier dynamical model. The model couples the Drude theory characteristics for probe beam transmission from equation (1) with the charge-carrier dynamics of equation (2). Mie theory simulations are seen in the figure insets.

It is apparent from the experimental and theoretical results of Fig. 4 that increasing sphere refractive indices yield increasing charge-carrier densities at the GaAs surface. This manifests itself by the observed polarities of the differential transmission curves. The negative curve of the *n*=1.51 sphere exhibits decreased probe transmission from pump-induced changes primarily to the absorption coefficient in the bulk—like that seen for the nominal semiconductor tests with a microscope objective. In contrast, the increasingly positive curves of the *n*=1.76, 1.83 and 1.98 spheres exhibit increased probe transmission from pump-induced changes primarily to the refractive index near the surface. For increasing sphere refractive indices, the preferential deposition of charge carriers near the semiconductor surface is also seen by way of reducing charge-carrier lifetimes. Charge-carrier lifetimes of *τ*=210, 120, 60 and 10 ps are measured for the sphere refractive indices of *n*=1.51, 1.76, 1.83 and 1.98, respectively. The decreasing charge-carrier lifetimes come about from a diminishing depth of focus and the corresponding preferential deposition of charge carriers at the semiconductor surface—where there exists a high surface state density and appreciable surface recombination velocity, *S*_v_=1.2 × 10^4^ m s^–1^. Curve-fitting of the Drude/charge-carrier dynamical model results to the experimental results confirms that the increasing sphere refractive indices reduce both the pump beam's photoinjection area, *A*_*φ*_, and photoinjection depth, *δ*_*z*_. For sphere refractive indices of *n*=1.51, 1.76, 1.83 and 1.98, the respective photoinjection depths into the semiconductor decrease, according to *δ*_*z*_=480, 300, 180 and 70 nm, and this leads to an increase in the initial charge-carrier densities.

Given these experimental findings, it is apparent that a nanojet focal geometry, with a sphere diameter of *d*=2.0 mm and refractive index of *n*=1.98, can support all-optical switching with femtojoule switching energies and picosecond switching times. The implementation tested here, with a GaAs target beyond the sphere, yields an ∼10-fJ switching energy (defined for a unity signal-to-noise ratio) and a 10-ps switching time. It is worth noting, however, that further reductions in the switching time can be met by enhancing surface recombination, and such enhancements are explored in the following section.

### The nanoparticle material system

Localized recombination is considered in this section, for enhanced AOS recovery, by introducing a semiconductor nanoparticle material system. The introduction of semiconductor nanoparticles increases the specific surface area to its highest theoretical limit, being *R*=12/*d* for a sphere. This maximization of surface-to-volume hastens the charge-carrier recombination rate, according to 1/*τ*=1/*τ*_b_+*S*_v_*R*. The charge-carrier lifetime, *τ*, can ideally be reduced to a value well below the bulk semiconductor lifetime, *τ*_b_.

The nanoparticle material system is integrated with the nanojet focal geometry, by coating nanoparticles over the entire surface of the dielectric sphere, to form the complete AOS architecture. An AOS architecture such as this enables ease of alignment, in that the geometry inherently overlaps the coincident pump and probe foci within the nanoparticles at the exit interface of the sphere, and it also enables omni-directionality, in that the coincident pump and probe beams can be incident over the full 4*π* steradians solid angle of the sphere.

The Si and SiC nanoparticles that are used have approximate diameters of 20 and 50 nm, respectively. (GaAs nanoparticles are not used, due to their high toxicity and limited knowledge for safe laser excitation and handling^30^,^31^.) The nanoparticles are coated according to the sample preparation steps outlined in the Methods section. The nanoparticle sizes are chosen to be an order of magnitude smaller than the pump and probe wavelengths, to minimize scattering. Moreover, the thickness of the nanoparticle coating is kept at or below the length of the photonic nanojet, to mitigate scattering of beams beyond the photonic nanojet. Scattering, if present, would be of greatest concern for the probe beam, as this beam must enter, exit and propagate well beyond the sphere, but it is found that the long wavelength of the 1,550-nm probe beam leads to minimal scattering^32^. Scattering for the 390 and 780-nm pump beams is also found to be negligible, given that the pump beam only propagates over the diameter of the nanoparticle-coated sphere, before it initiates switching of the probe beam.

The spheres used in the AOS architecture must be selected with careful consideration to their refractive index, *n*, and diameter, *d*. A high-intensity focus is required at the exit interface of the sphere, and this intensity depends on both *n* and *d*, according to Fig. 3. Spheres in the milli-scale regime, with diameters ranging from 0.1 to 2.0 mm, will simply require that the sphere have a refractive index of *n*≈2. (Such a range is defined for applicability and compatibility on the scale of optical fibres.) Spheres in the micro-scale regime, with diameters ranging from 1 to 30 μm, will require the sphere to have a refractive index along the blue curve of Fig. 3, between *n*≈1.75 and *n*≈1.83. (Such a range is defined for applicability and compatibility on the scale of network-on-chip devices.)

Given the considerations above for nanoparticles and dielectric spheres, the AOS architecture is tested with spheres in both the milli- and micro-scale regimes using nanoparticle coatings of both Si and SiC.

**Figure 5:**
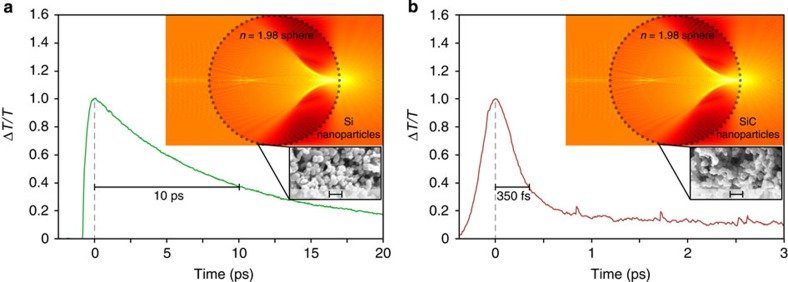
Milli-scale AOS architecture with Si and SiC nanoparticles. Experimental (positive) differential transmission for the probe beam, Δ*T/T*, is shown normalized versus time, for pump photoinjection of (**a**) Si nanoparticles and (**b**) SiC nanoparticles. The nanoparticles coat spheres with a diameter of *d*=2.0 mm and refractive index of *n*=1.98±0.02. The figures include insets with Mie theory simulations and SEM images of nanoparticles on a 200-nm scale.

Experimental results for the milli-scale AOS architecture are shown in Fig. 5. Figure 5a,b show results for Si and SiC nanoparticle-coated spheres, respectively. The spheres have a diameter of *d*=2.0 mm, being at the upper limit of the milli-scale regime, and are comprised of S-LAH79 glass, with a refractive index of *n*=1.98±0.02 The figure insets show a Mie theory simulation and scanning electron microscope (SEM) image of the nanoparticles.

Figure 5a shows the (positive) differential transmission, Δ*T*/*T*, for all-optical switching between pump and probe beams, with Si nanoparticles. The applied Si nanoparticles have a relatively low surface recombination velocity, *S*_v_=500 m s^–1^, but a noteworthy decrease in AOS recovery time is still observed—being approximately two thousand times faster than that of bulk Si. This rapid recovery is due to the promotion of surface recombination from the increased specific surface area of the nanoparticles. Ultimately, the milli-scale AOS architecture with the *d*=2.0-mm sphere and Si nanoparticles yields a switching time of 10 ps and switching energy estimated to be 200 fJ.

Figure 5b shows the (positive) differential transmission, Δ*T*/*T*, for all-optical switching between pump and probe beams, with SiC nanoparticles. The applied SiC nanoparticles have a relatively high surface recombination velocity, *S*_v_=4,000 m s^–1^, so a dramatic decrease in recovery time is observed—being approximately sixty million times faster than that of bulk SiC. (Detailed analyses and interpretations of the SiC charge-carrier dynamics are given in the Methods Section.) Ultimately, the milli-scale AOS architecture with the *d*=2.0-mm sphere and SiC nanoparticles meets the demands for all-optical switching, as it yields a switching time of 350 fs and switching energy estimated to be 100 fJ.

**Figure 6:**
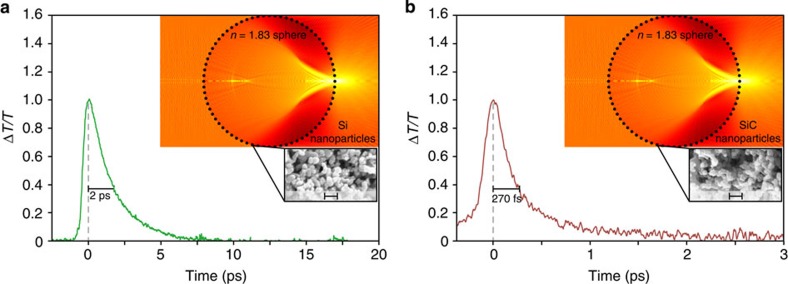
Micro-scale AOS architecture with Si and SiC nanoparticles. Experimental (positive) differential transmission for the probe beam, Δ*T/T*, shown normalized versus time, for pump photoinjection of (**a**) Si nanoparticles and (**b**) SiC nanoparticles. The nanoparticles coat spheres with a diameter of *d*=30–40 μm and refractive index of *n*=1.83±0.02. The figures include insets with Mie theory simulations and SEM images of nanoparticles on a 200-nm scale.

Experimental results for the micro-scale AOS architecture are shown in Fig. 6. Figure 6a,b show results for Si and SiC nanoparticle-coated spheres, respectively. The spheres have a diameter of *d*=30–40 μm, being at the upper limit of the micro-scale regime, and are comprised of N-LASF9 glass, with a refractive index of *n*=1.83±0.02 The figure insets show a Mie theory simulation and an SEM image of the nanoparticles.

Figure 6a shows the (positive) differential transmission, Δ*T*/*T*, for all-optical switching between pump and probe beams, with Si nanoparticles. The applied Si nanoparticles again produce a noteworthy decrease in the AOS recovery time—being even faster than that of the *d*=2.0-mm sphere in Fig. 5a. The speed enhancement for this micro-scale AOS architecture is attributed to its reduced focal spot area, compared with that of the milli-scale AOS architecture. The reduced focal spot area allows the micro-scale AOS architecture to avoid aggregates of larger, that is, 100^+^ nm, particles that are seen to be interspersed among the 20-nm nanoparticles in SEM images. The milli-scale AOS architecture, with its larger focal spot area is unable to avoid the aggregates of larger particles, which have a smaller specific area and longer charge-carrier lifetime, and this leads to its longer recovery time. Ultimately, the micro-scale AOS architecture with the *d*=30–40-μm sphere and Si nanoparticles yields a switching time of 2 ps and switching energy estimated to be 1 pJ.

Figure 6b shows the (positive) differential transmission, Δ*T*/*T*, for all-optical switching between pump and probe beams, with SiC nanoparticles. The applied SiC nanoparticles produce a dramatic decrease in AOS recovery time—being even faster than that of the *d*=2.0-mm sphere in Fig. 5b. The speed enhancement for this micro-scale AOS architecture is brought about from its reduced focal spot area, as stated above. Ultimately, the micro-scale AOS architecture with the *d*=30–40-μm sphere and SiC nanoparticles meets the demands for all-optical switching, as it yields a switching time of 270 fs and switching energy estimated to be 20 fJ.

## Discussion

An AOS architecture was introduced in this work. A guiding principle of localization was used in the design and analysis to facilitate AOS activation with femtojoule switching energies and AOS recovery with femtosecond switching times. The AOS architecture applied a nanojet focal geometry, in the form of a dielectric sphere, to focus high-intensity beams into a nanoparticle material system, in the form of a coating of semiconductor nanoparticles. The AOS architecture was implemented on milli-scale dimensions, with Si and SiC semiconductor nanoparticles, to yield respective switching energies of 200 and 100 fJ, with respective switching times of 10 ps and 350 fs. The AOS architecture was then implemented on micro-scale dimensions, with Si and SiC semiconductor nanoparticles, to yield respective switching energies of 1 pJ and 20 fJ, with respective switching times of 2 ps and 270 fs.

It was found that the AOS architecture could establish localized photoinjection of charge carriers, to enable femtojoule switching energies, and it could establish localized recombination of charge carriers, to enable femtosecond switching times. The AOS architecture met practical considerations for coupling, including beam alignment, directionality and capture cross-section, as well as practical considerations for stability, including physical and temperature sensitivity. Future applications of the AOS architecture may be implemented with consideration to coupling of the spheres to waveguides and/or fibres. Optical micro-electro-mechanical systems packaging can facilitate this integration, by way of on-chip spherical mounts based on V-grooves^33^,^34^, micropits^35^,^36^ or suspended microstructures^37^,^38^,^39^. Future applications of the AOS architecture may also include cascaded implementations, realized as daisy-chained spheres^40^, which are particularly challenging for AOS devices^41^, or parallel implementations^42^, which offer omni-directionality with multiple inputs/outputs. The proposed AOS architecture can be a building block for these future applications.

## Methods

### Experimental set-up

The pump–probe experimental configuration, seen in Fig. 1a, utilizes an erbium-doped fibre laser (Toptica Photonics, Inc.), with a 100-fs laser pulse duration, a 90-MHz repetition rate, and wavelengths of 780 and 1,550 nm for the respective pump and probe beams. The differential transmission, Δ*T*/*T*, of the probe beam is measured by time-resolved sampling as a function of the time delay between pump and probe pulses. The data are acquired with a lock-in amplifier (SR830) using a 100-ms time constant. The GaAs and Si targets use pump and probe pulses with respective wavelengths of 780 and 1,550 nm (that is, photon energies of 1.55 and 0.78 eV). The SiC target uses pump and probe pulses with respective wavelengths of 390 and 1,550 nm (that is, photon energies of 3.18 and 1.55 eV). The 780–1,550-nm pump–probe experiments with *d*=2.0-mm spheres use a pump–probe power ratio of 1:2. The 390–1,550-nm pump–probe experiments with *d*=2.0-mm spheres use a pump–probe power ratio of 1:20. The 780–1,550-nm pump–probe experiments with *d*=30–40-μm spheres use a pump–probe power ratio of 3:1. The 390–1,550-nm pump–probe experiments with *d*=30–40-μm spheres use a pump–probe power ratio of 1:1. For these pump–probe conditions, the pump photon energy is above-bandgap and the probe photon energy is below-bandgap. This leads to probe-power-independence for all reported results, that is, multi-photon contributions from the probe beam are not observed.

A tabletop pump–probe experimental set-up such as this characterizes the impulse response of the AOS architecture. For future applications, seeking compact device profiles and ultrashort laser pulses, the AOS architecture can be integrated with a sub-millimetre ultrashort pulsed laser, such as a mode-locked laser diode^43^, with the output response of such a device being a convolution of the impulse response measured in this work and the applied ultrashort laser pulse.

### Sample preparation

Experimental results are collected for focusing through spheres into a GaAs target and into nanoparticle coatings.

For the experiments that focus into a GaAs target, the spheres have a diameter of *d*=2.0 mm. They are mounted in the pump–probe experimental set-up by bonding the spheres to a 22-gauge needle tip with ultraviolet-curable polymer. The GaAs target is positioned in the focal plane of the spheres by collimating the back-reflected beams, as collimated back-reflected beams indicate that the reflective GaAs target is positioned in the focal plane of the sphere. Ultimately, the spheres with refractive indices of *n*=1.51, 1.76, 1.83 and 1.98 will be separated from the GaAs target by 480, 160, 100 and <1 μm, respectively. The respective sensitivities for these positions, being 1.1, 0.7, 0.6 and <0.5 μm, are dictated by each sphere's depth of focus.

For the experiments that focus into (Si or SiC) nanoparticle coatings, the spheres have diameters of *d*=2.0 mm and *d*=30–40 μm. The *d*=2.0-mm spheres are mounted as stated above. The *d*=30–40-μm spheres are mounted on a planar glass substrate. The nanoparticles are applied to the surface of the sphere with a dry-coating process^44^. It is found that the van der Waals forces in this process provide sufficient adhesion—although alternative nanoparticle dispersal and adhesion processes may be used, if needed, by way of polymer-based nanocomposite deposition^45^, chemical vapour deposition^46^ and/or laser ablation^32^. Unlike the tests of spheres with the GaAs target, the tests of nanoparticle-coated spheres are self-aligning, in that coincident pump and probe beams will inherently focus into the nanoparticle coatings.

### SiC charge-carrier dynamics

The bulk SiC results of Fig. 1b and the nanoparticle SiC results of Fig. 5b,b all exhibit AOS recovery with two distinct time constants. Long recovery time constants are seen at ∼22 μs for the bulk and at 5 and 1 ps for the SiC nanoparticles on the micro- and milli-scale architectures, respectively. The long recovery time constants are due solely to recombination on the charge-carrier density, *N*(*z*,*t*), and it is these time constants that witness pronounced effects from the increased specific surface area of the nanoparticles. Short-recovery time constants are observed at ∼3 ps for the bulk and at 350 and 270 fs for the SiC nanoparticles on the micro- and mill-scale architectures, respectively. The short-recovery time constants are attributed to ultrafast charge-carrier dynamics in the SiC bandstructure^47^, as photoinjected charge carriers populate excited states in the *M*-sidevalley and then undergo intervalley and intravalley scattering. The scattering yields femtosecond and picosecond transients for the effective mass and mobility. The effective mass and mobility transients form ultrafast transients on the refractive index near the surface, Δ*n*_s_(*t*), and absorption coefficient in the bulk, Δ*α*_b_(*t*), respectively, and this is ultimately seen as an ultrafast modulation on the differential transmission of the probe beam, according to equation (1). Such scattering processes have been observed before for SiC and commented on in the literature^39^,^48^,^49^.

## Additional information

**How to cite this article:** Born, B. *et al.* Integration of photonic nanojets and semiconductor nanoparticles for enhanced all-optical switching. *Nat. Commun.* 6:8097 doi: 10.1038/ncomms9097 (2015).
